# The Structural Modeling of the Interaction between Levofloxacin and the *Mycobacterium tuberculosis* Gyrase Catalytic Site Sheds Light on the Mechanisms of Fluoroquinolones Resistant Tuberculosis in Colombian Clinical Isolates

**DOI:** 10.1155/2014/367268

**Published:** 2014-04-28

**Authors:** N. Alvarez, E. Zapata, G. I. Mejía, T. Realpe, P. Araque, C. Peláez, F. Rouzaud, J. Robledo

**Affiliations:** ^1^Corporación Para Investigaciones Biológicas—(CIB), Bacteriology and Mycobacteria Unit, Carrera 72a N 78B-141, Medellín, Colombia; ^2^Universidad Pontificia Bolivariana—(UPB), Calle 78b No. 72a-109, Medellín, Colombia; ^3^Escuela de Ingeniería de Antioquia, Km 2 + 200 Vía al Aeropuerto José María Córdova, Envigado, Colombia; ^4^Grupo Interdisciplinario de Estudios Moleculares—(GIEM), Universidad de Antioquia, Calle 67 No. 108-53, Medellín, Colombia; ^5^Equal Opportunity Life Sciences, 1132 Parrish Drive, Rockville, MD 20851, USA

## Abstract

We compared the prevalence of levofloxacin (LVX) resistance with that of ofloxacin (OFX) and moxifloxacin (MFX) among multidrug resistant (MDR) *MTB* clinical isolates collected in Medellin, Colombia, between 2004 and 2009 and aimed at unraveling the underlying molecular mechanisms that explain the correlation between QRDR-A mutations and LVX resistance phenotype. We tested 104 MDR isolates for their susceptibility to OFX, MFX, and LVX. Resistance to OFX was encountered in 10 (9.6%) of the isolates among which 8 (7.7%) were also resistant to LVX and 6 (5.7%) to MFX. Four isolates resistant to the 3 FQ were harboring the Asp94Gly substitution, whilst 2 other isolates resistant to OFX and LVX presented the Ala90Val mutation. No mutations were found in the QRDR-B region. The molecular modeling of the interaction between LVX and the DNA-DNA gyrase complex indicates that the loss of an acetyl group in the Asp94Gly mutation removes the acid base interaction with LVX necessary for the quinolone activity. The Ala90Val mutation that substitutes a methyl for an isopropyl group induces a steric modification that blocks the LVX access to the gyrase catalytic site.

## 1. Introduction

According to reports from the World Health Organization (WHO), one-third of the world population is infected with* MTB* and about 10% develop the disease during their life. It is estimated that in 2011 there were 8.7 million new tuberculosis (TB) and 1.4 million deaths [1]. Even though effective drugs to treat TB have been available for more than 50 years, the absolute number of cases has continued to increase every year, as slow reductions in incidence rates continue to be outbalanced by increases in population [2]. Since 1993, the WHO has insisted on the practice of directly observed therapy (DOT) in which the patient is administered the medication by a health worker and observed taking it [3]. This measure was designed to not only increase treatment success rates but also to prevent further development of MDR-TB after the emergence of strains resistant to at least rifampin and isoniazid [4, 5]. However, insufficient government commitment, inadequate patient management, and public health policies as well as poor adherence to treatments and misuse of antibiotics have rendered MDR-TB a significant public health issue that poses a serious threat to global TB control [6]. As a result, the need for novel classes of anti-TB drugs has increased, with fluoroquinolones (FQ) becoming the drug of choice for second line use in MDR-TB treatment [7, 8] or in patients with intolerance to one of the first-line drugs [9]. Today the alarming current TB situation resides not only in its overall incidence but also in the emergence in 2006 of cases of extensively drug resistant (XDR)-TB [10] caused by* MTB* that, in addition of being MDR, are resistant to any FQ and to at least 1 of 3 injectable second line drugs amikacin, kanamycin, or capreomycin [1].

FQ to which OFX, MFX, and LVX belong are an important group of synthetic antibiotics that inhibit the bacterial DNA gyrase, thus inhibiting the DNA replication and transcription processes by preventing the ATP-dependent introduction of negative supercoils into closed circular DNA, as well as ATP-independent relaxation of supercoiled DNA [7, 11]. DNA gyrase is formed by GyrA and GyrB subunits which form a heterotetrameric A_2_B_2_ complex, the A and B subunits being encoded by the gyrase A (*gyrA*) and gyrase B (*gyrB*) genes, respectively [11]. The A subunit carries the active site for double-stranded DNA breakage and reunion, whereas the B subunit promotes ATP hydrolysis [12–14].

Studies have shown that amino acid substitutions occurring in the quinolone resistance, determining region of GyrA (QRDR-A) mainly clustered in codons 88, 90, 91, and 94, account for up to 96% of the mechanisms that confer FQ resistance in* MTB* [11, 15, 16]. In contrast, the substitution at position 95 in GyrA, which encodes a serine or threonine, has been shown to have no influence on FQ resistance [15].

Amino acid substitutions occurring in the quinolone resistance-determining region of GyrB (QRDR-B) are less related to FQ resistance and its implication in FQ resistance remains largely unclear. Among the 21 GyrB substitutions described in the literature, only two have been demonstrated to be implicated in resistance to FQ (N538D and E540V) [17].

It is known that the new FQ MFX and Gatifloxacin (GFX) have higher activity against* MTB* than LFX and their pharmacokinetic and pharmacodynamics properties make them an excellent alternative for treating MDR-TB cases [18]. However some studies have suggested that gatifloxacin may have more side effects that MFX and LVX such as glucose metabolism alterations [18].

LVX is an optical isomer of OFX and is characterized by its broad spectrum against gram positive, gram-negative bacteria, and other pathogens such as* Mycoplasma*,* Chlamydia*,* Legionella,* and* Mycobacteria* spp. Studies have reported that LVX is active against OFX resistant organisms including* MTB* and reaches high levels in the CSF as it can pass the blood brain barrier making it an excellent choice in cases of tuberculous meningitis [19].

The use of LVX in antituberculosis therapy presents certain advantages. The bioavailability of oral LVX is very rapid and complete, approaching 100%, and it is not affected when it is ingested after meals. LVX pharmacokinetics are similar during multiple-dose and single doses regimens and the pharmacokinetics are not appreciably affected by age, gender, or race when differences in renal function and body mass and composition are taken into account [19].

Colombia reported an overall incidence of 25–49 TB cases per 100,000 population for 2011 [1] and a proportion of MDR-TB among new TB cases of 2.4% between 2004 and 2005 [20]. However, the rate of MDR-TB among previously treated patients can reach more than 30%. Colombia is also one of the 84 countries until 2011 to have reported at least one case of XDR-TB [1]. Even though Colombia is not considered a high burden country for TB, current epidemiological data show that first line drugs as well as FQ resistance is a growing problem that needs to be urgently addressed. It even becomes alarming in the city of Medellin, an area of 2.4 million habitants out of which 681,000 (28.3%) live with less than 104 USD per month [21] and that has reported for 2011 an incidence of 78.9 TB cases per 100,000 population [22] with 175 MDR-TB cases since 2009 [23].

Our study first aimed to compare the prevalence of LVX resistance compared with resistance to OFL and MFX among MDR-TB clinical isolates collected between 2004 and 2009 in the metropolitan area of Medellin, Colombia, and establish a correlation with QRDR-A and QRDR-B mutations. Additionally, the study examined the structural interactions between the mutated GyrA enzyme and the LVX molecule using a homology modeling approach.

## 2. Materials and Methods

### 2.1. Isolates Selection and Mycobacterial Culture

A total of 104 MDR-*MTB *clinical isolates collected in Medellin, Colombia, between 2004 and 2009 and stored at −70°C at the Corporacion para Investigaciones Biológicas were used. These isolates had previously been tested for susceptibility to rifampin and isoniazid using a screening test in thin layer agar Middlebrook 7H11 (TLA 7H11) (Becton Dickinson) [24] and confirmed as MDR-TB using the Multiple Proportion Method in agar [25].

The MDR-*MTB* clinical isolates were grown in TLA 7H11 and incubated for 3 weeks at 37°C in a 5% CO_2_ atmosphere until luxuriant growth became apparent.

### 2.2. Fluoroquinolone Susceptibility Testing

Each of the 104 MDR-*MTB *isolates was tested for FQ susceptibility using the Multiple Proportion Method in agar.* MTB *colonies were first inoculated in liquid Middlebrook 7H9 medium and incubated for 2 to 3 weeks until MacFarland standard 1.0 was reached. Subsequently, serial 10^−2^ and 10^−4^ dilutions were prepared for inoculation into TLA 7H11 with antibiotics [24, 25]. OFX (Sigma-Aldrich), MFX (Kemprotec Ltd.), and LVX (Sigma-Aldrich) stock solutions were prepared at 1 mg/mL in water and stored at −70°C. The final concentrations that were used were OFX (2 *μ*g/mL), MFX (0.5 *μ*g/mL), and LVX (2 *μ*g/mL). Readings were performed weekly for 4 weeks and an isolate was considered resistant when the number of colonies on medium with the antibiotics was equal or greater than 1% of the number of colonies on the control media without antibiotics. The reference strain H37Rv* MTB *was used as a control.

### 2.3. *QRDR-A *and* QRDR-B *PCR Amplification

*MTB* isolates were cultured on TLA 7H11 for 3 weeks. Total DNA was isolated using the CTAB/NaCl method [26] and quantified using a NanoDrop ND-2000 spectrophotometer (Thermo Scientific) by measuring the absorption at 260 nm. Ten nanograms of DNA were used for PCR amplification using 2.5 U of the Fermentas Recombinant Taq DNA Polymerase (Thermo Scientific) and 60 pmol of each forward and reverse primer described in Table 1. The amplification was performed under the following conditions: 4 min at 94°C (1 cycle) followed by 30 s at 94°C, 30 s at 62°C, and 30 s at 72°C (40 cycles) and a final extension of 5 min at 72°C on a DNA Engine Thermal Cycler (Bio-Rad). DNA fragments corresponding to the QRDR of* gyrB *and* gyrA *were amplified under PCR conditions previously described in this methodology (Table 1).

### 2.4. Nucleotidic Sequences Analysis

The sequencing of the amplicons was outsourced and performed by Macrogen Korea (Seoul, Republic of Korea) with each strand being sequenced using a forward and a reverse primer. Two primers for* gyrA *and four for* gyrB *were used (Table 1). The chromatograms were analyzed and edited using FinchTV v 1.4 (http://www.geospiza.com/Products/finchtv.shtml) and the consensus sequences were established with ClonManager V.9.0 (Sdi-Ed Software). Nucleotidic sequence alignments were performed with ClustalW (http://www.ebi.ac.uk/Tools/clustalw2/index.html), and predictions of related aminoacid sequences were carried out with the ExPASy program (http://expasy.org/tools/dna.html).

### 2.5. Minimum Inhibitory Concentration (MIC)

OFX, MFL, and LVX minimum inhibitory concentrations (MIC) were determined for all isolates, and* MTB* H37Rv was used as a control. MIC were determined according to procedures previously described [25]. Briefly,* MTB* H37Rv and isolates were grown in Middlebrook 7H9 liquid medium until MacFarland standard 0.5 was reached. Cultures were homogenized on a minishaker using 4 mm glass beads. The FQ MIC assay was performed in Middlebrook 7H9 liquid medium in BACTEC MGIT 960. For each FQ, the concentrations tested were 0.25, 0.50, 1.00, 2.00, 4.00, 8.00, and 16.0 *μ*g/mL.

MGIT tubes were supplemented with 0.8 mL of supplement (BACTEC MGIT Growth Supplement; Becton Dickinson) and were inoculated with 0.1 mL of the drug solution and 0.5 mL of the test strain suspension. For preparation of the drug-free growth control tube, the organism suspension was diluted 100-fold in sterile saline solution, and then 0.5 mL was subsequently inoculated into the tube (susceptibility testing). The susceptibility testing sets were placed in the MGIT 960 instrument and results were interpreted as follows. At the time when the growth units (GU) of the drug-free control tube was >400, if the GU of the drug-containing tube to be compared was >100, the strain was Resistant. If the GU of the drug-containing tube was <100, the strain was susceptible [27].

### 2.6. Homology Modeling Analysis for QRDR-A

A homology modeling analysis was performed on* MTB *H37Rv and isolates TBR-67 and TBR-49 to determine the structural interactions between LVX and the gyrase A QRDR region. A search for identification of a three-dimensional structure of a protein known to serve as a model for determining the structure of the target protein was performed using the RCSB Protein Data Bank (PDB) accessible at http://www.pdb.org/pdb/home/home.do. A comparison was carried out using the* E. coli *DNA gyrase sequence (PDB access number 1AB4) and visualized with DeepView/Swiss-PdbViewer v3.7 (http://www.expasy.org/spdbv/). Additionally, the three-dimensional coordinates were read by Molecular RasWin Graphis Windows v2.6 (http://mc2.cchem.berkeley.edu/Rasmol/v2.6/).

The molecular characteristics of the GyrA catalytic site as well as the LVX chemical structure were determined using the semiempirical Austin Model 1 method which uses parameters derived from results of experimental quantum calculations to simplify and reduce computational costs [28]. Also, Austin Model 1 focuses on external electrons only considering that they are responsible for the reactivity. Models were optimized using global minimum geometry and energy parameters using PC Spartan Pro 1.0.5 (Wave function). The affinity values between the drug and the enzyme catalytic site were calculated for both the H37Rv reference strain and the two resistant isolates (TBR-49 and TBR-67) and the hydrogen bond length between the Asp94 and Arg98 residues and the LVX ionizable groups were also calculated using PC Spartan Pro 1.0.5 (Wave function).

## 3. Results

### 3.1. MDR Isolates FQ Susceptibility

Of the 104 MDR-TB isolates that were analyzed, 94 (90.4%) were sensitive to the 3 FQ tested. The remaining 10 (9.6%) were resistant to OFL, among which 8 (7.7%) were also resistant to LVX and 6 (5.7%) to MVX (Table 2).

### 3.2. QRDR-A and QRDR-B Mutations

Of the 10 (9.6%) isolates that were resistant to OFL, all were harboring the QRDR-A Ser95Thr substitution, a mutation also encountered in 88 (84.6%) isolates sensitive to the 3FQ (not shown). Four isolates (TBRs 67, 102, 111, and 176) which were all resistant to the 3 FQ were carrying the Asp94Gly mutation, while 2 (TBR-49 and TBR-31) which were resistant to only OFX and LVX had the Ala90Val substitution (Table 2). The 4 remaining isolates (TBR-73, 18, 103, and 107) did not harbor any mutation in positions 90 and 94 but carried the Ser95Thr substitution (Table 3). No mutations were found in the QRDR region of the* gyrB *gene.

### 3.3. Minimum Inhibitory Concentration (MIC) of FQ Resistant Isolates

The MIC for OFL, MFX, and LVX were determined for the 10 FQ resistant isolates (Table 3). The highest MIC for OFL (16 mg/L) were encountered in 2 isolates harboring the Asp94Gly substitution (TBR-67 and TBR-111); however TBR-102 and TBR-176 also harboring the same mutation showed a MIC of 8 mg/L. The 2 isolates bearing the Ala90Val mutation both had a MIC of 4 mg/L and the MIC for the isolates with no QRDR-A mutation were equally distributed between 4 mg/L and 8 mg/L. For LVX, the MIC were spread between 2 mg/L and 8 mg/L with no particular trend in the distribution. For MFX, TBR-49 and TBR-31 both had a MIC of 0.5 mg/L. The highest MIC (2 mg/L) was measured in isolates with either no QRDR-A mutation (TBR-73) or the Asp94Gly substitution. Within 10 FQ resistant* MTB* isolates were found 6 XDR (Table 2).

### 3.4. Modeling of the Interaction between LVX and GyrA

We hypothesized an acid-base interaction between LVX and the GyrA catalytic site in which the amino acids 90, 94, and 98 have been previously shown to be directly involved in the antibiotic fixation.

**Figure 1:**
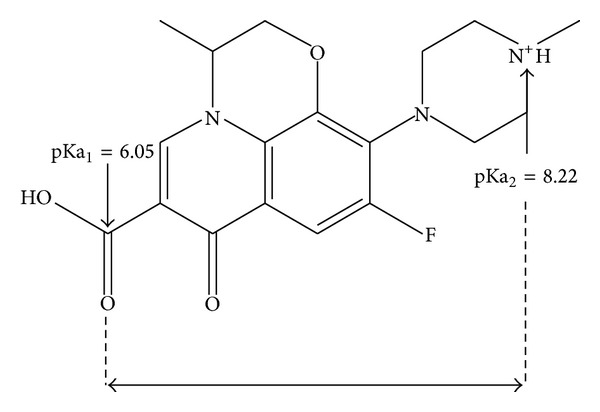
Structural representation of the LVX molecule with its ionizable groups.

**Figure 2:**
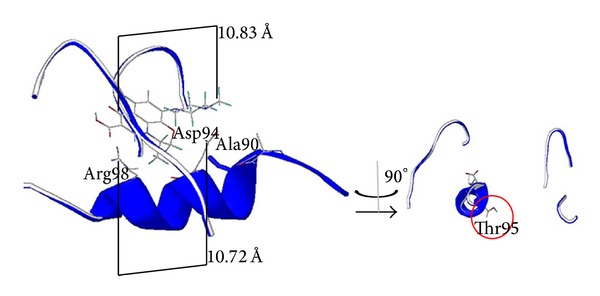
Model representation of the interaction between LVX and H37Rv* MTB* GyrA catalytic site featuring interatomic distances.

We established a first model using the H37Rv reference strain in which we calculated that the interatomic distance between the Asp94 and Arg98 residues was 10.72 Å. This distance between both amino acids correlates with the distance between LVX ionizable groups (10.83 Å) and is therefore thought to allow enough space for LVX to interact (Figures 1 and 2).

**Figure 3:**
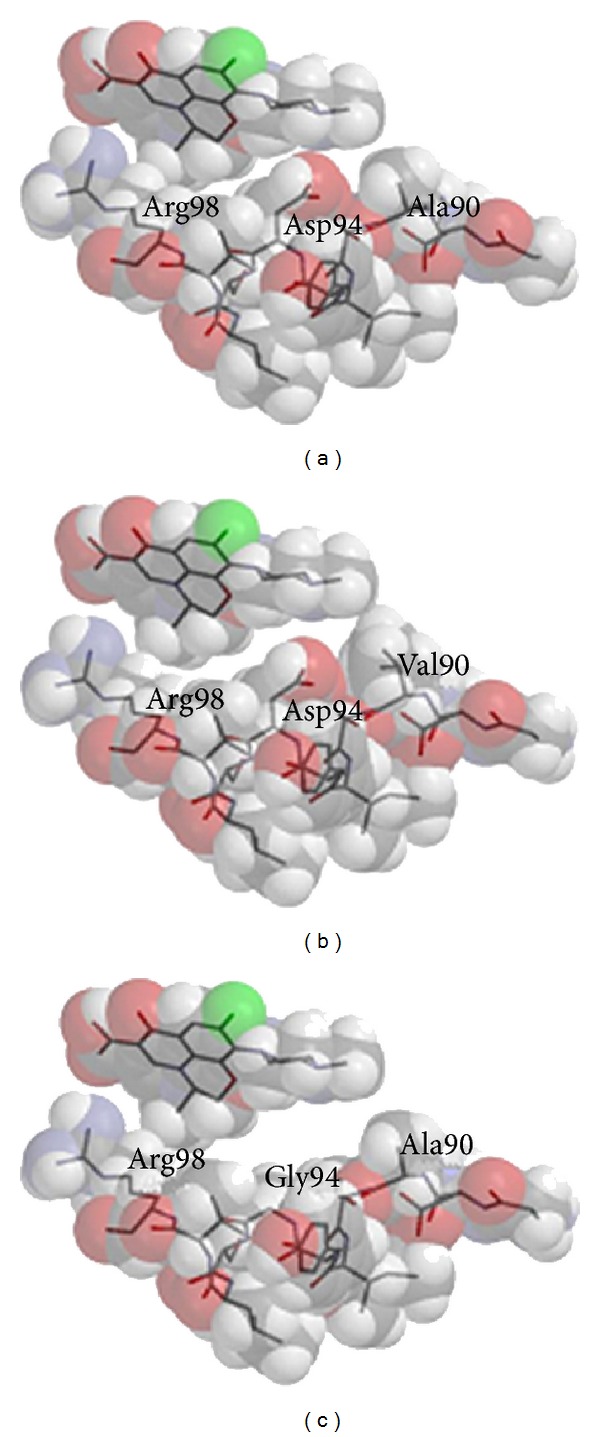
Comparative** s**tructural three-dimensional model representation of the interaction between LVX and* MTB* GyrA catalytic site in H37Rv (3(a)), TBR-49 (3(b)), and TBR-67 (3(c)).

Taking the interatomic distances into consideration a structural representation of the interaction between LVX and the H37Rv GyrA catalytic site was performed (Figure 2), showing the nonimplication of the Thr in position 95. The Ser95Thr substitution in TBR-49 and TBR-67 did not affect the interaction between LVX and GyrA catalytic site (Figures 2 and 3).

The Ala90Val substitution in TBR-49 resulted in an increase of the amino acid electron density volume from 83.88 Å^3^ to 119.62 Å^3^ due to the substitution of a methyl group by an isopropyl group (Figure 3(b)). This generated a steric hindrance impairing LVX interaction with GyrA catalytic site.

The TBR-67 Asp94Gly generated the loss of an acid (acetyl) group, thus preventing the acid-base interaction between the LVX amino group and the Asp acid group at the catalytic site (Figure 3(c)).

In addition the affinity values between the drug and the enzyme catalytic site were calculated for both the H37Rv reference strain and the two resistant isolates (TBR-49 and TBR-67), and we found that the more stable configuration was given for the H37Rv-LVX complex with an interaction energy of −970.83 kcal/mol. For both strains with* gyr*A mutations we found that the interaction energies were −955.83 kcal/mol and −866.48 kcal/mol for the TBR-49 (Ala90Val)-LVX and the TBR-67 (Asp94Gly)-LVX complexes, respectively. The more negative the affinity value (kcal/mol) between the drug and the enzyme is, the more stable the interaction complex is. Also, we calculated the hydrogen bond length between the Asp94 and Arg98 residues and the LVX ionizable groups (Table 4). Interestingly, the distance between the Arg98 residue and LVX is greater for the TBR-49 (Ala90Val) isolate (5,143 Å) due to the steric effect generated by increasing the volume density when an isopropyl group is added to the catalytic site. For the TBR −67 isolate (Asp94Gly) the loss of the acetyl group prevents the interaction with the LVX amino group, consequently nonhydrogen bond could be calculated.

## 4. Discussion

MDR-TB is an increasing global problem as it has now been identified in almost every country and continues to climb in many parts of the world with alarmingly high levels as in some areas, one in five (19%) TB patient is infected by an MDR-TB strain [1]. The situation is even more dramatic in retreatment cases for which the prevalence is up to 60% [29, 30].

FQ broad spectrum of antibacterial activities and convenient use have made them a drug of choice for empiric therapy of a variety of common infections, such as urinary tract, upper and lower respiratory tract, enteric and gonococcal infection, even sometimes when a causative organism has not been identified. With the expanded use of these broad-spectrum agents for many infections, the selective pressure applied onto microbial pathogens has resulted in the emergence of FQ resistant strains in a diversity of organisms, including* MTB *[31].

Since the 1990s, the incidence of FQ-resistant* MTB *has been gradually increasing first with ciprofloxacin and OFX, and today with MFX and LVX [32]. The impact of FQ resistant TB as well as the critical need to control it and understand its mechanisms is underscored by the rapid emergence and spread of XDR-TB [33]. High MDR-TB burden countries located in Eastern Europe and Central Asia reported XDR-TB in more than 10% of MDR-TB cases. To date, a cumulative total of 84 countries (Colombia reported its first XDR-TB case in 2009) have confirmed at least one case of XDR-TB.

In this study, we endeavored to examine the resistance to MFX, OFX, and LVX of 104 clinical isolates of MDR* MTB* that had been collected in Medellin, Colombia, between 2004 and 2009. Those isolates had been phenotypically characterized and stored at the Mycobacterial Unit of the Corporación para Investigaciones Biológicas. However, we had no data indicating whether they were new or retreatment cases isolates. We encountered an FQ resistance rate of 9.6% which is comparable with the one described by other recent studies in Latin America [34] but far below the prevalence reported in Russia [35]. FQ resistance in Colombia is scarcely documented and in fact, to our knowledge, this is the 1st study reporting numerical data on this particular issue. The 10 isolates that displayed FQ resistance were further tested for the injectable amikacin and capreomycin as well as for ethionamide and para-aminosalicylic acid (PAS) and 6 of them (5.8%) (TBR-102, 111, 176, 18, 107, and 103) were found to be XDR (data not shown). This XDR prevalence among MDR strains is comparable to what has been reported in other regions of Latin America [36].

It has been widely reported that the majority of FQ-resistant* MTB *isolates carry mutations in the QRDR part of the* gyrA *gene [35, 37, 38]. However, the mutation we encountered the most frequently is known to be a natural polymorphism unrelated to resistance [39]. The Ser95Thr substitution is carried by 88 susceptible and the 10 resistant isolates.

Substitution in GyrA codons 90 and 94 have often been linked to FQ resistance [37, 40]. Of the 8 isolates resistant to the 3 FQ, 4 carried the Asp94Gly substitution (TBR-67, 102, 111, and 176) and 2 carried the Ala90Val mutation (TBR-49 and 31) (Table 2). None of the resistant isolates harbored both mutations unlike what was reported in several studies [15, 41]. Recently Nosova and colleagues showed that isolates carrying the double mutations exhibit higher MICs, strongly suggesting a synergistic contribution of both substitutions. Two isolates (TBR-73 and TBR-107) did not harbor any mutation in positions 90 and 94, indicating that additional GyrA independent mutations or mechanisms could also confer FQ resistance. Mutations in GyrB such as Asn538Asp, Asp500His, and Asp495Asn have been shown to be associated with* MTB *cross-resistance to FQ [35, 42], but in this study no mutations in QRDR-B region were found. On the other hand, TBR-18 that resist to OFL and MXF but not to LVX do not carry any mutation in position 90 or 94 indicating that if OFX and MFX resistance can be expressed without those substitutions, susceptibility never occurs in their presence. Additionally this shows that OFX and MFX resistance can be expressed independently of that of LVX.

It is important to clarify that all the isolates that were sensitive to LVX or MFX had a MIC below the cutoff (2.0 *μ*g/mL), except for the TBR-107 isolate, with a MIC in the cutoff value for LVX (2.0 *μ*g/mL), but was truly sensitive to MFX (<0.25 *μ*g/mL). Other resistance mechanisms such as those involving the action of efflux pumps may explain the phenotypic FQ resistance in* MTB* without mutations in* gyrA* or* gyrB* genes. There are studies that show that the overexpression of efflux pumps from the ABC family can be correlated with expulsion of fluoroquinolones and a concomitant MIC increase. Further studies are necessary to assess the role efflux pumps in phenotypic resistance to FQ of* MTB* clinical isolates [43].

We determined MICs for the 10 FQ tested (Table 3). Even though there was no proper correlation between MIC levels and any of the mutations, the isolates harboring the Asp94Gly substitution tend to resist higher FQ concentrations. The stronger effect of Asp94Gly corresponds to a trend previously suggested by several studies [35, 44].

In order to better understand the mechanisms of LVX resistance conferred by substitutions in positions 90 and 94 and study them at the atomic level, we established a three-dimensional molecular model of the interaction between the hypothesized structure of the GyrA QRDR region and LVX. We used the same homology modeling technique that had allowed Cunha and colleagues to propose the first* MTB *GyrA hypothetical structure three-dimensional model [13]. Calculating the distance between Asp94 and Arg98 facilitated the visualization of a pocket that is believed to accommodate LVX (Figures 1 and 2), thus underscoring the critical role played by both residues in the FQ binding. LVX is a chiral fluorinated carboxyquinolone that possesses 2 ionizable functional groups, a carboxylic moiety (pKa_1_ = 6.05) and a basic piperazinyl group (pKa_2_ = 8.22) [45] that are critical to explain the acid-base interaction with GyrA catalytic site (Figure 1).

The Ala90Val change generated the addition of an isopropyl group at the catalytic site inducing an electron density volume increase (Figure 3(b)) and a steric effect that reduces the interaction between the LVX amino group and GyrA. Studies in* E. coli *suggest that a DNA gyrase that possesses such mutations is not affected in its function of DNA breakage [46] and maintains its functionality.

In the case of the Asp94Gly substitution, that acid-base interaction is hampered by the loss of an acetyl group (Figure 3(c)) thus rendering LVX binding less efficient and providing resistance to the drug by target modification. Modifying the enzyme does prevent LVX action; however it has been shown to have no effect on DNA binding, therefore maintaining a fully active gyrase [46].

Different studies have demonstrated the presence of individuals within the same* MTB* population causing infection in the same patient and expressing different susceptibility to drugs. This phenomenon is known as heteroresistance and has been described as present in 25% of isolates resistant to OFX [47]. Although the presence of heteroresistance was not assessed in this study, we cannot totally exclude this phenomenon without further experiments.

FQ are actually the more active drugs in the treatment of MDR-TB; nevertheless, it is still unknown many aspects related to resistance mechanisms including those nonassociated to mutations. As FQ resistant TB continues to develop into more alarming and threatening public health issue, gaining structural information on the resistance mechanisms can facilitate the rational engineering for improving the actual drugs. The homology modeling approach has become a promising tool that can provide a deeper insight of antibiotic-target interaction essential to the design of much needed new molecules.

## 5. Ethical Considerations

This project is classified in the category “research without risk” according to the Helsinki Declaration. For this study applies only experimental techniques of laboratory to isolates of MTB and at no time were biological, physiological, psychological, and social interventions in humans performed.

## Figures and Tables

**Table 1:** Primers used to amplify and sequence QRDR-A and QRDR-B regions.

Target region	Primer	Nucleotide sequence	Method
QRDR-A	gyrA15F	5′-GATGACAGACACGACGTTGC-3′	PCR
	gyrA19R	5′-GCCAGCTCACGCAGGTTG-3′	
	gyrA17F	5′-ATCGACTATGCGATGAGCGTG-3′	Sequencing
	gyrA18R	5′-ATGCCGCCTGACCCGTTG-3′	

QRDR-B	gyrB5F	5′-ACCTTCGCCAACACCATCAACACC-3′	PCR and sequencing
	gyrB10R	5′-CGAACCGAGGGATCCATGGTG-3′	
	gyrB7F	5′-CGGTTCTGCAAAAAGCGGTCGC-3′	Sequencing
	gyrB8R	5′-CGGAAGTATCGCCTGGAACATCG-3′	

**Table 2:** QRDR-A and QRDR-B mutations spectrum for the 10 FQ resistant *MTB* isolates.

Isolates	SNP	Amino acid change	Codon	Susceptibility to FQ		
				OFX	LVX	MFX
TBR-67*	GAC/GGC	Asp/Gly	94	R	R	R
TBR102*(XDR)	GAC/GGC	Asp/Gly	94	R	R	R
TBR-49*	GCG/GTG	Ala/Val	90	R	R	S
TBR-111*(XDR)	GAC/GGC	Asp/Gly	94	R	R	R
TBR-31*	GCG/GTG	Ala/Val	90	R	R	S
TBR-176*(XDR)	GAC/GGC	Asp/Gly	94	R	R	R
TBR-73*	No mutation			R	R	R
TBR-18*(XDR)	No mutation			R	S	S
TBR-107*(XDR)	No mutation			R	S	S
TBR-103*(XDR)	No mutation			R	R	R

*Isolates with Ser95Thr mutation.

R: resistant isolate; S: susceptible isolate.

**Table 3:** OFL, LVX, and MFX MIC for ten isolates fluoroquinolones resistant X: last dilution with growth.

Isolate	Mutation in GyrA	Mutation in GyrB	OFL MIC mg/L							Cut-off MIC for resistance	LVX MIC mg/L							Cut-off MIC for resistance	MFX MIC mg/L							Cut-off MIC for resistance
			0.25	0.5	1	2	4	8	16		0.25	0.5	1	2	4	8	16		0.25	0.5	1	2	4	8	16	
TBR-49	Ala90Val	NM					X			>2.0						X		>2.0		X						>0.5
TBR-67	Asp94Gly	NM							X							X					X					
TBR-73	NM	NM						X								X						X				
TBR-31	Ala90Val	NM					X								X					X						
TBR-103	NM	NM						X							X						X					
TBR-18	NM	NM					X							X						X						
TBR-107	NM	NM					X							X					<0.25							
TBR-111	Asp94Gly	NM							X							X						X				
TBR-102	Asp94Gly	NM						X								X						X				
TBR-176	Asp94Gly	NM						X							X						X					

**Table 4:** Interaction energy and hydrogen bond length between GyrA and LVX.

Complex	Interaction energy	Hydrogen bonds (Arg98-LVX)	Hydrogen bonds (Asp94-LVX)
H37Rv-LVX	−970.83 kcal/mol	4.778 Å	2.599 Å
TBR-49-LVX	−955.83 kcal/mol	5.143 Å	2.673 Å
TBR-67-LVX	−866.48 kcal/mol	4.500 Å	—
